# Insights into insecticide-resistance mechanisms in invasive species: Challenges and control strategies

**DOI:** 10.3389/fphys.2022.1112278

**Published:** 2023-01-09

**Authors:** Junaid Ali Siddiqui, Ruidong Fan, Hira Naz, Bamisope Steve Bamisile, Muhammad Hafeez, Muhammad Imran Ghani, Yiming Wei, Yijuan Xu, Xiaoyulong Chen

**Affiliations:** ^1^ College of Agriculture, College of Tobacco Science, Guizhou University, Guiyang, China; ^2^ International Jointed Institute of Plant Microbial Ecology and Resource Management in Guizhou University, Ministry of Agriculture, China & China Association of Agricultural Science Societies, Guizhou University, Guiyang, China; ^3^ Guizhou-Europe Environmental Biotechnology and Agricultural Informatics Oversea Innovation Center in Guizhou University, Guizhou Provincial Science and Technology Department, Guiyang, China; ^4^ Research and Development Centre for Fine Chemicals, National Key Laboratory of Green Pesticides, Guizhou University, Guiyang, China; ^5^ Department of Entomology, South China Agricultural University, Guangzhou, China; ^6^ Henry Fok School of Biology and Agriculture, Shaoguan University, Shaoguan, China; ^7^ State Key Laboratory of Rice Biology, Institute of Insect Sciences, Zhejiang University, Hangzhou, China; ^8^ Guangxi Key Laboratory of Rice Genetics and Breeding, Guangxi Crop Genetic Improvement and Biotechnology Lab, Rice Research Institute, Guangxi Academy of Agricultural Sciences, Nanning, China; ^9^ College of Science, Tibet University, Lhasa, China

**Keywords:** biological invasion, insecticide resistance, biodiversity risk, exotic species, resistance mechanisms

## Abstract

Threatening the global community is a wide variety of potential threats, most notably invasive pest species. Invasive pest species are non-native organisms that humans have either accidentally or intentionally spread to new regions. One of the most effective and first lines of control strategies for controlling pests is the application of insecticides. These toxic chemicals are employed to get rid of pests, but they pose great risks to people, animals, and plants. Pesticides are heavily used in managing invasive pests in the current era. Due to the overuse of synthetic chemicals, numerous invasive species have already developed resistance. The resistance development is the main reason for the failure to manage the invasive species. Developing pesticide resistance management techniques necessitates a thorough understanding of the mechanisms through which insects acquire insecticide resistance. Insects use a variety of behavioral, biochemical, physiological, genetic, and metabolic methods to deal with toxic chemicals, which can lead to resistance through continuous overexpression of detoxifying enzymes. An overabundance of enzymes causes metabolic resistance, detoxifying pesticides and rendering them ineffective against pests. A key factor in the development of metabolic resistance is the amplification of certain metabolic enzymes, specifically esterases, Glutathione S-transferase, Cytochromes p450 monooxygenase, and hydrolyses. Additionally, insect guts offer unique habitats for microbial colonization, and gut bacteria may serve their hosts a variety of useful services. Most importantly, the detoxification of insecticides leads to resistance development. The complete knowledge of invasive pest species and their mechanisms of resistance development could be very helpful in coping with the challenges and effectively developing effective strategies for the control of invasive species. Integrated Pest Management is particularly effective at lowering the risk of chemical and environmental contaminants and the resulting health issues, and it may also offer the most effective ways to control insect pests.

## 1 Introduction

Invasive species pose risks to human health, food security, the survival of endangered species, economic loss, and ecosystem stability (Venette et al., 2021). Invasive pest species are also known by the names non-native, exotic, non-indigenous, and introduced. The human race is responsible for its introduction, whether on purpose, by accident, or through trade. Besides the obvious danger to biodiversity, these introduced species also pose a significant risk to national biosecurity (Rao et al., 2018; Raghuteja et al., 2022). Species can now be transported to new regions of the world at an extraordinary pace due to the rapid expansion of the world’s commercial and social networking. Awareness of the negative effects of invasive species on local biodiversity and the resulting economic loss in many nations has grown over the past two decades (Pimentel et al., 2005; Zhang et al., 2022). Recent research into the evolutionary genetics of invasive species has found that the ability to adapt to natural selection may be more important for the successful invasion of some introduced species than widespread physiological plasticity or tolerance. Therefore, it can unintentionally boost the invasive species’ evolutionary potential and enable its quick growth and geographical distribution in the area of introduction (Lavergne and Molofsky, 2007; Marbuah et al., 2014; Siddiqui et al., 2021; Venette et al., 2021; Hafeez et al., 2022b).

As arthropods spread outside of their natural habitats, they meet naïve animals and host plants as well as population-controlling pathogens, parasitoids, and predators. Opportunities to dominate and modify invaded areas arise when antagonistic and coevolutionary relationships among invasive species are disrupted. The first introduction of invasive species (Grape phylloxera pest, *Daktulosphaira vitifoliae*) was reported in Europe about the middle of the 19th century. At the same time, the emerald ash borer, *Agrilus planipennis*, landed in North America and Europe in the early twenties. The fall armyworm, *Spodoptera frugiperda*, devours maize in Africa, causing a direct impact on food or the environment (Sileshi et al., 2019), Darwin’s finches in the Galapagos were under attack by invading flies (*Philornis downsi*) (Fessl and Tebbich, 2002; Koop et al., 2021), or red turpentine beetle (*Dendroctonus valens*) has a devastating effect on China’s pine forests (Sun et al., 2013). Indirect effects can be more subtle, such as when the brown marmorated stink bug (*Halyomorpha halys*) spreads an aflatoxin-producing fungus in the United States (Opoku et al., 2019), or In South America, dengue fever is spread by the Asian tiger mosquito (*Aedes albopictus*) (Ricas Rezende et al., 2020), and *Xylella fastidiosa*, a non-native bacteria, is spread *via* spittlebugs (Occhibove et al., 2020). Indirect consequences on water, health, and the environment may result from measures taken in response to arthropod invasions, particularly if widespread use of pesticides is required (Milano and Chèvre, 2019).

Each year, the world’s food supply faces a 10%–30% loss due to insect pests (Oerke, 2006) and exotic species-related damages worth $20 billion (Pimentel et al., 2000). That is why it is so important to keep the pests that have affected our economy under control (Savary et al., 2012). Insecticide use is thought to be the most effective pest control technique. Pesticides are heavily used in managing the pest in the current era. Consequently, one billion kilos of chemicals are used annually to combat crop pests (Alavanja, 2009; Drees et al., 2013). In several countries, pesticide usage has increased, i.e., Britain (18,000 tons), Italy (62,000 tons), Germany (4,800 tons), Poland (2,400 tons), and China (273,375 tons) (FAOSTAT Food and Agriculture Organization of the United Nations, 2020). Despite their effectiveness, synthetic pesticides have raised questions about resistance to pests, environmental degradation, and potential health impacts are all reasonable concerns (Du et al., 2020).

Insecticides are used to eliminate pests and disease-carrying insects in farming. Several insecticides, including organophosphates, pyrethroids, and neonicotinoids, play pivotal roles in pest management (Abdulahi et al., 2011). One of the most prominent examples of micro-evolution is the development of pest resistance due to the widespread usage of insecticides. More than 500 distinct pest species have been documented as having developed resistance to insecticides, according to previous research (Connor et al., 2011). Numerous pests, like corn earthworms and others, feed on a wide variety of crops around the world (cotton, peanuts, tobacco, *etc.*) and have developed resistance to insecticides. Several elements, biological, genetic, and operational, contribute to the emergence of resistance, but genetic aspects are regarded as the most helpful (Karaağaç, 2012). However, future populations showing increasing insecticide resistance make pest control more difficult (Stratonovitch et al., 2014). Insects have been found to use a variety of molecular resistance mechanisms, including metabolic and target sites.

Understanding species-level worldwide patterns are also helpful from a biosecurity aspect. Resistance-containing species pose a significant concern as intruders as they can build new populations that are already evolved to insecticidal stress. Invasive populations of the Silverleaf white fly, *Bemisia tabaci*, are just one example of a historical problem in Australia (Thia et al., 2021). Similarly, after the introduction of western flower thrips, Franklieniella occidentalis, into Australia, many of the chemical pesticides used were ineffective against the insect (Reitz and Funderburk, 2012). However, numerous urban pests are resistant to pesticides (e.g., cockroaches, houseflies, mosquitoes, ants, wasps, and termites) (Acevedo et al., 2009; Liu, 2015; Mahapatro, 2017; Fardisi et al., 2019). Even though insects can negatively affect agriculture, public health, and the environment, effective methods of controlling them are lacking (Hardy, 2014).

Cumulative global knowledge of resistance mechanisms helps develop hypotheses and expectations at more localized scales. The current review literature beam spotlights available knowledge on invasive species and broadly expands on the various species that have been documented to develop a broad spectrum of resistance against various classes of chemical insecticides. Moreover, possible mechanisms of insecticide resistance in invasive species, their management strategies, and future implication are also discussed.

## 2 Resistance in invasive insects

Pesticide-induced resistance is defined as a heritable modification in a pest population’s sensitivity to pesticides, as evident in the repeated failure of the treated chemical products to achieve the required control (Mahapatro, 2017). Resistance studies are essential in developing strategies for resistance mitigation and reducing possible insect pest outbreaks. A large number of invasive insect species have been examined and found to exhibit resistance to various classes of insecticides in China (Table 1), and in some other parts of the world (Table 2).

**TABLE 1:** A list of insecticide-resistant invasive species of China.

Scientific name	Common names	Resistance against insecticide	Country	References
*Aedes albopictus*	Asian tiger; mosquito	Pyrethroid; Fenthion; Glyphosate; deltamethrin	China	Hou et al. (2020); Chen et al. (2018)
*Bactrocera cucurbitae*	Dacus cucurbitae	Organophosphorus; pyrethroid	China	Gu et al. (2015)
*Bactrocera dorsalis*	Oriental fruit fly	Malathion; Beta-cypermethrin, cyhalothrin	China	Wang et al. (2011); Chen et al. (2015)
*Bemisia tabaci*	Cotton white fly	Bifenthrin; thiamethoxam; acetamiprid; imidacloprid; chlorpyrifos	China	Qiu et al. (2009); Wang et al. (2011); Yang et al. (2014)
*Blattella germanica*	German cockroach	Organophosphorus; DDVP; fenitrothion; diazinon; pirimiphosmethyl; chlopyrifos; propoxur; bendiocarb	China	Pai et al. (2005)
*Cydia pomonella*	Codling moth	Chlorpyrifos; carbaryl	China	
*Eriosoma lanigerum*	Woolly apple aphid	imidacloprid	China	Jing et al. (2016)
*Frankliniella occidentalis*	Western flower thrips	Cyhalothrin; spinosad	China	Wang et al. (2011); Wang et al. (2014)
*Leptinotarsa decemlineata*	Potato beetle	Neonicotinoid	China	Liu et al. (2011); Xiong et al. (2010)
*Liriomyza sativae*	Vegetable leaf miner	Chlorpyrifos	China	Yan et al. (1998)
*Lissorhoptrus oryzophilus*	Rice water weevil	Chlorphenamide	China	Liu et al. (2018)
*Pectinophora gossypiella*	Pink bollworm	Decamethrin	China	Li et al. (1995)
*Periplaneta americana*	American cockroach	Neonicotinoid	China	Zhang et al. (2018)
*Solenopsis invicta*	Red imported fire ant	Fipronil, beta-cypermethrin, indoxacarb	China	Siddiqui et al. (2022, 2022a)
*Spodoptera frugiperda*	fall armyworm	Indoxacarb	China	Hafeez et al. (2021, 2022a)
*Trialeurodes vaporariorum*	Greenhouse whitefly	Malathion; glyphosate; deltamethrin; neonicotinoid; pyrethroids	China	Zhang et al. (1980); Liu et al. (2005)
*Thrips palmi*	Melon thrips	Organochlorine; Organophosphorus; Pyrethroids	China	Zhao et al. (1995); Immaraju et al. (1992); Broadbent and Pree (1997)
*Trogoderma granarium*	khapra beetle	Phosphine	China	Borah et al. (1981)

**TABLE 2:** A list of insecticide-resistant invasive species in the world.

Scientific name	Common names	Resistance against insecticide	Country	References
*Bactrocera dorsalis*	Oriental fruit fly	Organophosphorus	Pakistan	Khan and Akram (2018); Hsu et al. (2004); Vontas et al. (2011)
		Carbamates	Taiwan	Khan and Akram (2018); Hsu et al. (2004); Vontas et al. (2011)
		Pyrethroid,; spinosad; Trichlorfon	unknown	Khan and Akram (2018); Hsu et al. (2004); Vontas et al. (2011)
*Bemisia tabaci*	Cotton white fly	Parathion-methyl; endosulfan	America	Byrne and Devonshire (1993)
		Imidacloprid	Europe	Wang et al. (2011)
*Cydia pomonella*	Codling moth	Arsenate; DDT; organophosphorus; benzoyl urea	America	Hough (1928); Cutright (1954); Moffit et al. (1988); Welter et al. (1991)
		Decamethrin; abamectin	France	Bouvier et al. (1998); Reyes and Sauphanor (2008)
		Glutathion; Chlopyrifos; Phosalone	Spanish	Rodríguez et al. (2010)
*Frankliniella occidentalis*	Western flower thrips	Carbamates (Methiocarb, Bendiocarb)	Australia	Martin and Workman (1994); Espinosa et al. (2002); Herron and James (2005); Götte and Rybak (2011)
		Organochlorine	New Zealand	Martin and Workman (1994); Espinosa et al. (2002); Herron and James (2005); Götte and Rybak (2011)
		Organophosphorus	Spain	Martin and Workman (1994); Espinosa et al. (2002); Herron and James (2005); Götte and Rybak (2011)
		Pyrethroid (fenvalerate)	United States of America	Martin and Workman (1994); Espinosa et al. (2002); Herron and James (2005); Götte and Rybak (2011)
*Leptinotarsa decemlineata*	Potato beetle	Carbofuran; pyrethroid	Canada	Harris and Svec (1981)
*Periplaneta americana*	Americana cockroach	Imidacloprid	America	Wang et al., 2004,2006; Ko et al., 2016
*Blattella germanica*	German cockroach	Fipronil	America	Wang et al., 2004,2006; Ko et al., 2016
*Periplaneta australasiae*	Australian cockroach	Abamectin	America	Wang et al., 2004,2006; Ko et al., 2016
*Spodoptera frugiperda*	fall armyworm	Cyhalothrin; flubendiamide; chlorantraniliprole	America	Gutiérrez-Moreno et al. (2019); Yu (1991)
*Thrips palmi*	Melon thrips	Organochlorine; Organophosphorus	America	Zhao et al. (1995); Immaraju et al. (1992); Broadbent and Pree (1997)
		Pyrethroids	Canada	Zhao et al. (1995); Immaraju et al. (1992); Broadbent and Pree (1997)

### 2.1. Mechanism of insecticide resistance

There is various mechanism mediating insecticide resistance development in insects. The major factors are behavioral resistance, fitness cost, reduced penetration, target resistance, and metabolic resistance. The mechanism underlying insecticide resistance in insects can generally be categorized as follows.

1) Behavioral resistance
2) Fitness cost
3) Penetration resistance
4) Target-site resistance
5) Metabolic resistance
6) Resistance-inducing operational factors

#### 2.1.1 Behavioral resistance

**FIGURE 1:**
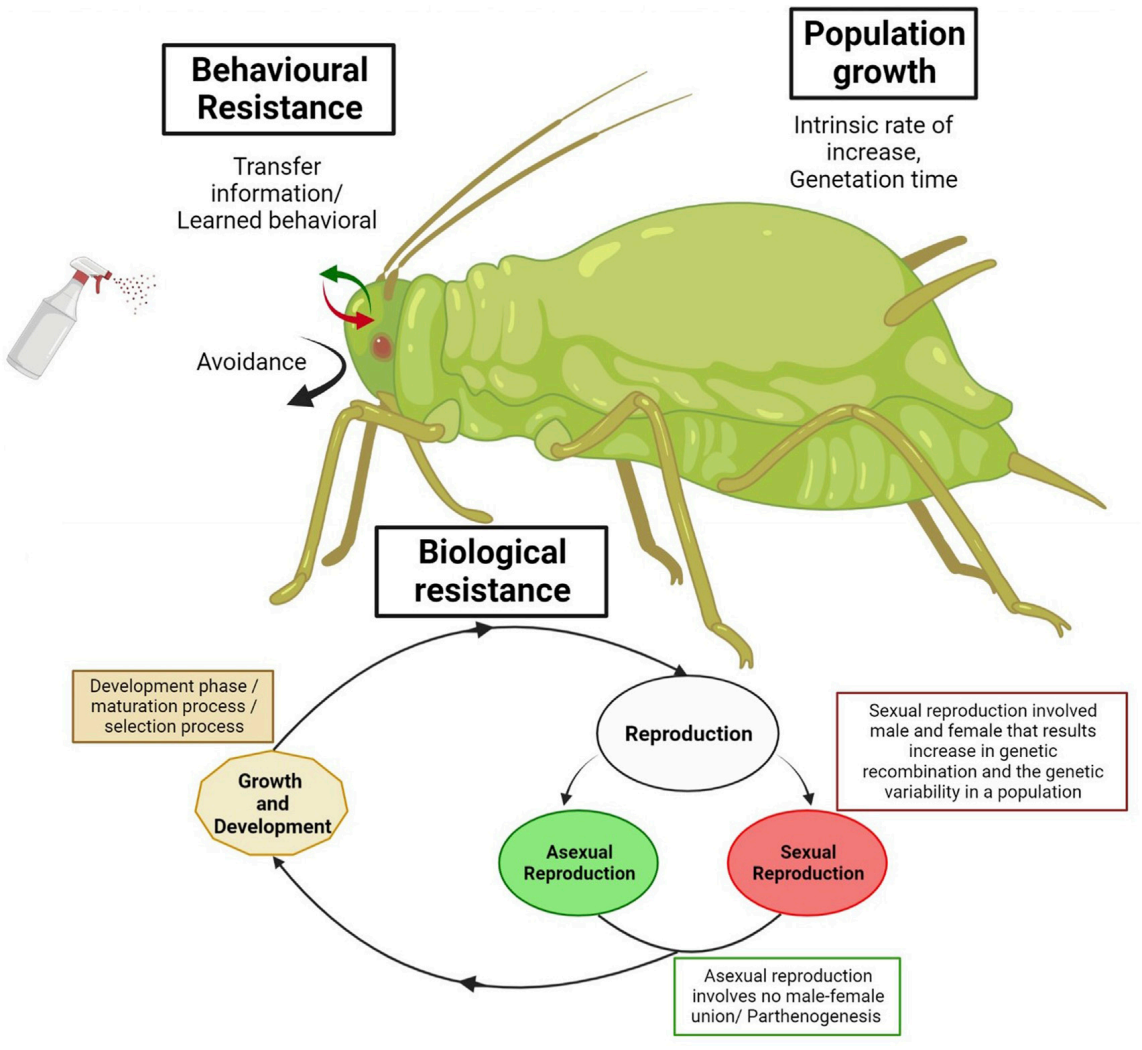
Schematic diagram of the biological and behavioral mechanisms of insecticide resistance in invasive insects.

In the very first line of protection, organisms might develop strategies to lessen their exposure to the uptake of a pesticide (Dunlop et al., 2018; Lushchak et al., 2018). Insects can develop resistance to chemicals through a number of different mechanisms, but an early and important one is a behavior response (Nansen et al., 2016) (Figure 1). When exposed to a lethal toxin, insects may often cease feeding and may even leave the treated area by simply moving from one field to another or into a deeper crop canopy (De Roode and Lefèvre, 2012; IRAC, 2022).

Behavioral resistance was well defined in a previous study by Sparks et al. (1989) as “evolved behavior that reduces an insect’s exposure to toxic compounds or that allows an insect to survive in what would otherwise be a toxic and fatal environment”. To survive, arthropod pests use behavioral processes, which involve shifts in behavior and stay away from areas that have been sprayed with insecticides (Sparks et al., 1989). Insect movement can have a significant effect on the growth of insecticide resistance. Movement characteristics of an insect cannot determine the extent of insecticide exposure, impacting the selection pressure on the insect population. It can also determine the degree to which insect populations mix in the ecosystem, thus affecting the ability of resistant ales to increase in resistance. For instance, the Colorado potato beetle (*Leptinotarsa decemlineata*) has limited flight capabilities, so pockets of insecticide resistance have developed in growing regions across North America (Alyokhin et al., 2008). For instance, *Plutella xylostella* Linnaeus, an invasive species, has developed behavioral resistance by using site selection for egg laying in adults and larval mobility variations to escape lethal dosages of foliar-applied insecticides in the field (Sarfraz et al., 2005; Zago et al., 2014). da Silva Nunes et al. (2020) found variation in the locomotor activity of *P. xylostella* adults between laboratory and field-collected samples. Insects collected from the field exhibited elevated activity and decreased resting time compared to controls, suggesting that they were motivated to escape from treated surfaces. Host plant evasion may have resistance traits, such as producing more eggs or preferring to oviposit on plants with less hairy and delicate cuticles, as evidenced by research demonstrating these insects have evolved to avoid insecticides (Silva and Furlong, 2012; Ang et al., 2014). Some other insects, such as female sheep blowflies (*Lucillia cuprina*), developed the ability to delay ovulation in the presence of a pesticide intended for their larvae (Mariath et al., 1990).

Red Imported Fire Ants (RIFA) are the best example of behavioral resistance because their excellent social system benefits their success (Dhami and Booth, 2008; Bertelsmeier et al., 2017; Jones and Robinson, 2018; Giraldo et al., 2021). Various behaviors play a role in developing resistance in RIFA, such as antibiotic secretions and adaptive immunological responses of colony members (Wilson-Rich et al., 2009), self- and mutual grooming by ant nestmates can restrict or accelerate infection and toxin transmission (Theis et al., 2015). Moreover, the effective waste removal of unhealthy materials (including dead ants). The ants regularly clean and disinfect each other and the infected ants they come into contact with (Pull et al., 2018). Examples of behavioral resistance occurred in recent studies, Wen et al. (2020) reported that invasive fire ant (*Solenopsis invicta*) deposited soil particles on the ant replant to avoid contact. To minimize pesticide contact, another study found that RIFA carried debris particles to cover the pesticide-treated region (Wen et al., 2021). When social ants come into touch with previously immunized nestmates, their ability to resist infection significantly improves (Konrad et al., 2018). This social transfer of infection resistance could explain how colony members’ survival rates are raised due to group life, despite the higher risks of transmission of alien agents (pesticides) that occur (Traniello et al., 2002). Pesticide resistance has been recently found in RIFA, which might be the reason for the inability to manage urban invasive ant species (Allen et al., 2018). The ineffectiveness of numerous biological and chemical control programs to control the invasive species could be attributed to a lack of understanding of ant behavior and the habits mentioned above, among several other factors.

#### 2.1.2 Fitness cost

##### 2.1.2.1 Biological resistance

The biological and ecological factors discussed here constantly interact to impact the risk of resistance development. Life cycle and population factors are important biological parameters.

Life cycle: An overriding aspect of insect biology that impacts an insect’s ecological interactions and the development of pesticide resistance is the insect’s life cycle (Sudo et al., 2018). Through a pest’s life cycle, there are changing interactions with its host and environment. Optimal resistance management practices rely on an understanding of these interactions. The life cycle of the insect pest is the primary factor affecting the development of insecticide resistance; in particular, the long life processes in bugs (Saulich, 2010) and short life cycle with the abundant progeny of mosquitoes which has all the properties suitable to swiftly developing resistance (Karunamoorthi and Sabesan, 2013).

The insects have different reproductive phases that influence resistance development through behavioral and genetic effects. Moreover, some ecological factors, such as environmental or host quality, will affect the reproduction phase (Helps et al., 2017). Most insects undergo sexual reproduction, involving a male and female union, and the resulting genetic recombination increases the genetic variability in a population. For example, sexual reproduction in two-spotted spider mites (*Tetranychus urticae*) enhanced the genetic variability in its population. It allowed for an increased potential for the development of resistance (Sun J. et al., 2022). On the other hand, asexual reproduction involves no male-female union, with the best example found in aphids (Stöck et al., 2021) (Figure 1). This will increase the reproduction rate, but it will also limit the genetic variability in a population. Multiple generations of asexually reproducing aphids, such as the green peach aphid (*Myzus persicae* Sulzer), are produced each year. Still, only a single sexual generation is likely to occur (Guillemaud et al., 2003). Among these reproductive phases, the insects undergo the development phase, which is a maturation process. Organisms vary a great deal in how these occur. Selection pressure can often occur throughout this life cycle phase. For example, the Housefly goes through distinct development stages (e.g., egg, pupa, adult fly) as it grows (de Jonge et al., 2020). Moreover, the sweet potato white fly, *B. tabaci*, has been shown to be resistant to neonicotinoids. The effects of thiamethoxam resistance selection on the life histories of *B. tabaci* B-biotype strains were studied by comparing those of selected and non-selected strains over multiple generations (Feng et al., 2009). The resistant individuals had shorter life spans and lower fecundity than the susceptible unselected strain. On the other hand, the nymphal stages took longer to mature in the resistant strain. Further phenotypic changes were seen in the resistant strain, with reduced body size across all instars and pupal stages compared to the susceptible strain (Feng et al., 2009). So, the life span of the insect pest, its reproductive capacity, and its surrounding habitat are crucial elements in the evolution of resistance (Naqqash et al., 2016). An insect’s lifecycle and developmental period play a crucial part in the growth of insecticide resistance (Figure 1).

Population growth: The growth of insect populations influences resistance development by determining the most successful individuals. Intense selection pressures from insecticide use, short generation time, and readily available host crops have resulted in diamondback moth (*P. xylostella*) resistance to almost all groups of insecticides (Taha, 2022; Venkatesan et al., 2022). The intrinsic rate of increase is the rate that an insect population will increase without external constraints. When populations are affected by biological or environmental constraints, the growth rate slows as it approaches a carrying capacity (large population size that is sustainable) (Holt, 2009). Insect populations are regulated by multiple factors, e.g., environmental conditions, host quality, and natural enemies. In the example shown, insect predator populations lag behind prey populations, but prey population decline after predator populations build up (Culshaw-Maurer et al., 2020). The invasive insects invade a new place where they do not have a natural enemy, and the environmental condition is suitable for their survival, consequently, reasons for their success. For example, *Solenopsis invicta* invades many countries, can increase their population, and dominates the local fauna (Morrison et al., 2004; Siddiqui et al., 2021) (Figure 1).

Generation time is also important in the time required for individuals to complete their life cycle. This ranges from multiple years per life cycle, as in the case of large pine weevil (*Hylobius abietis*) (Inward et al., 2012), to numerous life cycles per year in Mosquitoes (Mendoza, 2016). This allows for more genetic recombination, increasing genetic diversity and the probability of selecting resistant alleles. The population with multiple generations per year would more likely develop resistance to a pesticide because there would be more selection cycles (chances) to select for or to raise the percentage of resistant ones in the final population. For example, mosquitoes have multiple generations in short periods, which increases the possibility of the transfer of resistance alleles to the next generations and increases their genetic diversity, leading to the resistant generation (Mendoza, 2016). Another invasive species, Diamondback moths, can undergo multiple generations annually, increasing the potential for selection pressure across the population (Kliot and Ghanim, 2012) (Figure 1).

#### 2.1.3 Penetration resistance

**FIGURE 2:**
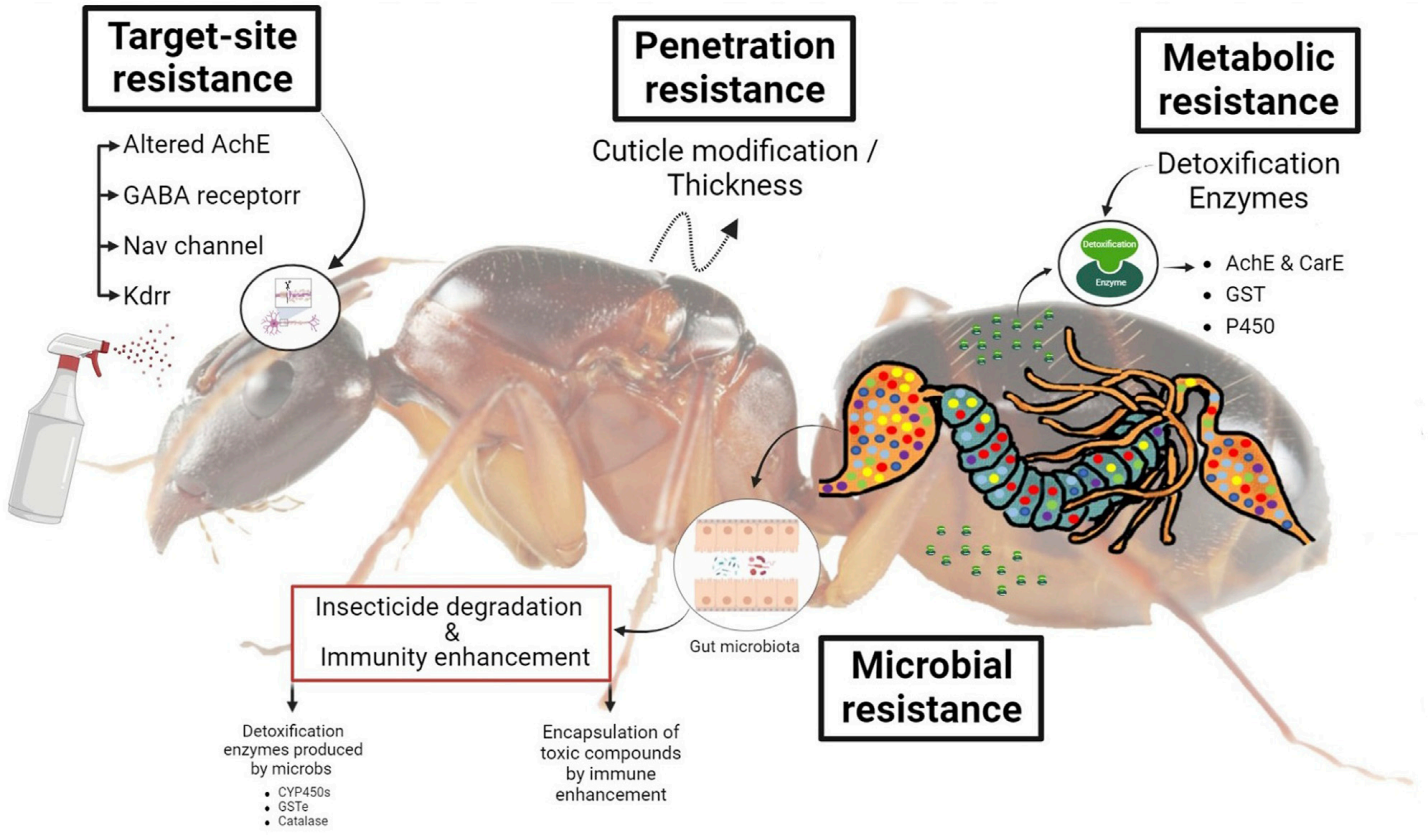
A schematic diagram represents the role of enzymes and gut microbiota in pesticide detoxification in invasive ants.

The insect cuticle comprises two major layers of polysaccharide chitin, lipids, and proteins. The chitin is in the inner procuticle, but there is no chitin in the thin outer epicuticles (Bass and Jones, 2016). By coating cuticular hydrocarbons (CHCs) generated in particular secretory cells in the epidermis called oenocytes on their epicuticle, insects are able to protect themselves from drying out (Falcon et al., 2019). Penetration resistance occurs when insects slow down the engagement of xenobiotics within their bodies. Insects create barriers against the product using their outer cuticle, which protects them against a wide spectrum of insecticides (Figure 2) (Tangtrakulwanich and Reddy, 2014; Balabanidou et al., 2016). Because of this slowing process, insecticides may take significantly longer to reach their protein targets in neuronal cells (Fang et al., 2015). Scanning electron microscopy indicated that resistant mosquitos have significantly increased cuticle thickness, with the outer epicuticle accounting for much of the entire difference in whole cuticle thickness (Balabanidou et al., 2016). Some adaptations known as penetration mechanisms slow the rate at which a pesticide is absorbed through the cuticle (Gunning et al., 1991; Ahmad et al., 2006). Evidence of penetration experimentation is frequently used to infer resistance (Ahmad et al., 2006; Puinean et al., 2010); however, in order to distinguish expression differences in cuticle-related genes between resistant and susceptible strains, transcriptomics has also been applied (Thia et al., 2021). However, minimal studies on cuticular resistance in invasive insects such as *P. xylostella* revealed a significant difference in cuticular microRNAs between the chlorantraniliprole resistance population and the susceptible population. They give insight into cuticular alteration during the development of resistance (Zhu et al., 2017; Shabbir et al., 2021). There is evidence of cuticular resistance in *H. armigera*, which successfully prevented deltamethrin adsorption in the body by generating thicker cuticles in resistant individuals compared to susceptible individuals, where insecticide percolation was substantially higher (Ahmad et al., 2006). According to one study on *Amyelois transitella* (Lepidoptera: Pyralidae), when an insect is repeatedly subjected to pesticide pressure, the hydrocarbon profile in the cuticle tends to change, which may help to build cuticular resistance (Ngumbi et al., 2020).

Thickening or altering the chemical makeup of the insect cuticle reduces toxicant uptake. A similar mechanism was observed in OP-resistant strains of *Culex tarsalis* (Whyard et al., 1994) and *C. quinquefasciatus* (Gong et al., 2022). The variation in pesticide transport across the cuticle of houseflies (*Musca domestica* Linnaeus) was also demonstrated (Malik et al., 2007). Similarly, organophosphates, methyl carbamates, pyrethroids, and neonicotinoids resistance in *Tribolium castaneum* (Rösner et al., 2020), OP resistance in Colorado potato beetle (*Lepinotarsa decemilineata*) (Yoon et al., 2022), and fenvalerate resistance in *P. xylostella* (Mubashir and Seram, 2022), have all been confirmed to be partly due to reduced penetration (Figure 2). This mechanism confers low levels (less than 5 - fold) of resistance and only becomes important when combined with other resistant mechanisms. On the other hand, it appears to shield several different classes of pesticides (Venkatesan et al., 2022). Comparing the amount of pesticide taken over time by resistant and susceptible types of insects is the simplest technique to calculate the penetration rate. Although, even a slight reduction in penetration might contribute significantly to the invasive insect developing resistance to the insecticide (Venkatesan et al., 2022).

#### 2.1.4 Target-site resistance

Insecticides are chemicals (synthetic compounds or direct biological materials) used to control insect pests (Wojciechowska et al., 2016). However, the most successful synthetic insecticides are neuro-inhibitors (Figure 2). Despite the increasing cost and the risk of environmental contamination, these are the world’s most widely used agents of insect pest control today (Yadav et al., 2022). Our study mainly discussed commonly used four classes of organic or synthetic pesticides, and they are as follows: organochlorines, organophosphates (OPs), carbamates, and pyrethroids (Table 3). All of these pesticides target the insect’s central nervous system. For organochlorines, the neurological system is the primary target. The inhibitory neurotransmitter gamma-aminobutyric acid (GABA) attaches to a specific type of chloride channel called a GABA-gated chloride channel, and blocking these channels leads to increased excitability in neurons (Al-Kuraishy et al., 2022). The enzyme that breaks down acetylcholine, a neurotransmitter, is the target of carbamates and OPs. Other organochlorines and pyrethroids inhibit nerve impulse transmission by binding to and blocking the function of Nat channel proteins in the neuron membrane (Lopez-Suarez et al., 2022). Pyrethroids affect the sodium channels and lead to paralysis of the organism. When they attach to sodium channels, they trigger excitatory paralysis and eventually death in insects (Davies et al., 2007).

**TABLE 3:** Insecticide classification based on target site.

Chemical group	Insecticide	Target site
Organochlorines	chlordane, dieldrin, DDT, lindane, methoxychlor, kepone, toxaphene, mirex, and benzene hexachloride	GABA- gated chloride channel blocker
Organophosphates	Azinphos-methyl, azamethiphos, fenitrothion, diazinon, dichlorvos, phosmet, parathion, tetrachlorvinphos, malathion, methyl parathion, chlorpyrifos, terbufos	Acetylcholinesterase (AchE) inhibitors
Carbamates	Aldicarb, fenobucarb, oxamyl, carbofuran, ethienocarb, carbaryl, and methomyl	Acetylcholinesterase (AchE) inhibitors
Pyrethroids	alpha-cypermethrin, deltamethrin, and permethrin	Sodium channel modulators (Nav) Pyrethroids

See http://www.irac-online.org/modes-of-action/

Biologically modified insects may be resistant to insecticides because they have been altered genetically to inhibit the insecticide from binding or interacting at its site of action (Fenibo et al., 2022). The second most researched resistance mechanism in a wide variety of insects is target site insensitivity. As a result of changes in the target site’s structure or accessibility, toxicants may no longer form stable bonds with them, a phenomenon known as target-site insensitivity (Venkatesan et al., 2022). Aphids have developed a resistance to pyrethroids by developing resistance at the target site (knockdown resistance-kdr) (Valmorbida et al., 2022).

There are four major types of target sites such as 1) The insensitivity of acetylcholine esterase (AChE) has been identified as an essential mechanism for pesticide resistance in numerous agricultural insect pests to carbamates and organophosphates (Renault et al., 2022). Esterases are a diverse family of enzymes responsible for breaking down a wide range of ester bonds in both endogenous and exogenous substrates (Memarizadeh et al., 2011) and play important roles in the insect’s ability to eliminate the toxic effects of insecticides. The enzyme acetylcholinesterase (AChE) is the target of both organophosphate and carbamate insecticides because of its central role in the nervous system in hydrolyzing cholinergic neurotransmitters and terminating nerve impulses (Devrnja et al., 2022). Neonicotinoids, organophosphates, and methyl carbamates act as acetylcholinesterase inhibitors or as nicotinic acetylcholine receptor agonists (Devrnja et al., 2022). Prior to inhibiting acetylcholinesterase (AChE), organophosphorus insecticides must be transformed into their oxon analogs by monooxygenases (Lorke and Petroianu, 2019) (Figure 2). 2) Sodium channel blocker insecticides (SCBIs) are a relatively new group of pesticides exemplified by metaflumizone and indoxacarb, which are commercially registered chemicals. It is well-known that SCBIs like pyrethroids and DDT intoxicate insects by blocking their access to voltage-gated sodium channels (VGSCs) (Cens et al., 2022). The sodium channel in nerve cells is disrupted by KDR (knockdown resistance). This mechanism is widely exploited in pyrethroid and DDT resistance. Kdr and super kdr are the results of a number of different mutations (Sun H. et al., 2022). 3) For insects, the nicotinic acetylcholine receptors (nAChRs) play a crucial role in learning and memory due to their participation in fast neurotransmission (Taillebois and Thany, 2022). Resistance to imidacloprid has been widely investigated in *B. tabaci*, *Aphis gossypii*, and *M. persicae*, due to modifications in and subunit of nAChR (Xu et al., 2022; Zhou et al., 2022). Synthetic insecticides like neonicotinoids and spinosad aim to target nAChRs because of their essential role in insect neurotransmission (Kaleem Ullah et al., 2022). d) The GABA receptor is primarily a Cl—channel, and GABA receptor molecules are essential to target locations for multiple chemically diverse kinds of pesticide-active chemicals, resulting in fipronil, cyclodienes, and avermectins resistance (Burman et al., 2022; Venkatesan et al., 2022).

#### 2.1.5 Metabolic resistance

Metabolic resistance is a type of resistance inferred by metabolic activities in insects that help them detoxify or break down contaminants or the ability to eliminate toxic compounds from their bodies more quickly (Venkatesan et al., 2022). Insect metabolism is critical in the development of pesticide resistance against various groups of chemical pesticides, including carbamates, organophosphates, and synthetic pyrethroids. Insects metabolize insecticides to less toxic or non-toxic forms *via* a mechanism called “detoxification” (Jaffar et al., 2022). Metabolic resistance is of huge importance and is also one of the most studied mechanisms in insects. Insects use their enzymatic systems to digest pesticides, and resistant populations may have more of these enzymes or enzymes with improved detoxifying capabilities (Karunaratne and Surendran, 2022). Furthermore, to become more effective, these enzyme systems may also be able to break down many different types of pesticides (Vyas et al., 2022). Detoxifying enzymes enhance and accelerate the drug resistance caused by the metabolism of insecticides (Siddiqui et al., 2022c; Siddiqui et al., 2022b). The over-produced enzymes in pests can develop protection against insecticides (Figure 2) (Rane et al., 2016; Khan et al., 2020).

##### 2.1.5.1 Carboxylesterases (CarEs)

Carboxylesterases (CarEs) are an adaptable class of lipolytic enzymes that catalyze the hydrolysis of esters into alcohol and acid molecules. Widespread biocatalysis, drug metabolism, and endobiotic and xenobiotic degradation rely on these enzymes (Sood et al., 2016). CarEs enzymes also detoxify environmental toxicants such as pyrethroids, a major insecticide class used worldwide (Staudinger et al., 2010). Recent studies on invasive species indicated that a greater AChE and CarE esterase activity was seen in resistant populations compared to vulnerable ones such as *S. invicta* (Siddiqui et al., 2022b; 2022c), *H. armigera* (Young et al., 2005; Wu et al., 2011), *C. tarsalis* (Whyard et al., 1994), and *Aonidiella aurantia* (Grafton-Cardwell et al., 2004) (Figure 2).

Biochemical methods using *S. litura* resistant strains from Korea and India have shown the role of acetylcholinesterases in pesticide resistance (Yonggyun et al., 1998; Kranthi et al., 2002; Muthusamy et al., 2011). In the same way, a carbamate-resistant strain of *S. exigua* from California had an AChE enzyme that was about 30 times less sensitive to methomyl than the enzyme from a sensitive laboratory strain (Byrne and Toscano, 2001). However, molecular information on the AChE point mutation exists for a number of species of *Spodoptera*, while it is mostly available for *S. frugiperda*. When comparing two strains of this species, Yu et al. (2003) found that acetylcholinesterase from a field strain taken from Florida corn fields was up to 85-fold less responsive to inhibition by CBs and Ops (Hilliou et al., 2021).

The CarE activity reported by various studies, such as alpha and beta esterase production, when exposed to fipronil, was greater in a resistant strain of the mosquito (*Culex quinquefasciatus*) than in a susceptible strain (Sarkar et al., 2009). Increased activity of CarE was detected in beta-cypermethrin-resistant *M. domestica* when compared to beta-cypermethrin-susceptible ones (Zhang et al., 2007). Moreover, elevated esterases activity was reported after insecticides exposure to the peach potato aphid (*M. persicae*) (Bass and Field, 2011; Venkatesan et al., 2022) and the brown planthopper (*Nilaparvata lugens*) (Tang et al., 2022). This means that elevated esterases can detoxify the insecticides. Gene amplification is the cause of the increased production of these enzymes (Şengül Demirak and Canpolat, 2022). In mosquitoes, amplified esterase molecules are more reactive with insecticides than non-amplified esterases (Gan et al., 2021).

Insecticide detoxification by CarE has also been reported in other insect species. For example, some previous studies have indicated an increase in the activity of CarE in honeybees following exposure to fipronil (Carvalho et al., 2013; Roat et al., 2017). Similarly, the grassland locust, *Epacromius coerulipes* Ivanov (Orthoptera: Acrididae), showed a significantly higher CarE activity in a strain that had evolved resistance against fipronil than in a susceptible strain (Jin et al., 2020). Insecticide metabolism studies indicated that esterases are involved in resistance mechanisms. Increased accumulation of esterase hydrolytic products in insecticide-resistant insects compared to susceptible gives evidence for the involvement of these enzymes (Jensen, 2000; Silva et al., 2012; Khan et al., 2021b; Siddiqui et al., 2022b).

##### 2.1.5.2 Glutathione S-transferases (GST)

Glutathione transferases (GSTs) are a broad and diversified family of enzymes that are present in almost all organisms. GST is a class of multifunctional proteins that are extremely important in the biological transformation of exogenous compounds, drug metabolism, and protection from peroxidation (Hollman et al., 2016; Dasari et al., 2018). It was discovered that the house fly *M. domestica* contains DDT-dehydrochlorinase as a glutathione S-transferase (Clark, 1989). They are crucial for the detoxification of both internal and external substances, intracellular movement, the generation of hormones, and the prevention of oxidative stress (Hassan et al., 2019). GST is one of the foremost detoxifying enzymes of insecticides in insects. Insecticides can be metabolized either by reductive dehydrochlorination, which is facilitated by GSTs, or by conjugation reactions with reduced glutathione, which result in water-soluble metabolites that are more easily eliminated. Additionally, they help to remove dangerous oxygen-free radical species that are formed as a result of the use of insecticides (Panini et al., 2016). Neonicotinoid resistance in the *B. tabaci* is correlated with constitutive overexpression of several ESTs, GSTs, UGTs, CYPs, and ABC transporters (Vassiliou et al., 2011) (Figure 2).

Several major insecticide types, such as boric acid, carbamates, organophosphorus, organochlorine, and pyrethrin-resistant in German cockroaches, have increased their GST expression levels to varying degrees (Gentz and Grace, 2006; Nasirian, 2010; Rinkevich et al., 2013). Studies on invasive fire ants show that exposure to indoxacarb, beta-cypermethrin, and fipronil causes a substantial alteration in GST activity. Additionally, the outcomes demonstrate that population resistance and sublethal concentrations substantially impact enzyme activity (Siddiqui et al., 2022c; Siddiqui et al., 2022b). OP and DDT resistance mechanisms were reported in houseflies (Ranganathan et al., 2022). In a resistant strain of *M. domestica*, Zhang et al. (2007) discovered that GST activity was also markedly elevated. According to Sarkar et al. (2009), the GST activity of all populations tested was greater than that of a susceptible laboratory strain. Studying orthopterans, researchers found that a particular strain of *E. coerulipes* had much higher GST activity than susceptible insects (Jin et al., 2020). Insecticide treatments for maize rootworms have already been linked to an increase in GST activity, according to a recent study (Souza et al., 2020). Pest resistance has already been reported in different insects against DDT and OP.

##### 2.1.5.3 Cytochrome P450 monooxygenases (CYP or cytochrome P450)

An essential supergene family, cytochrome P450 (P450 or CYP), is responsible for the metabolism or attenuation of toxicity of numerous potentially harmful substances (Zhu et al., 2017; Hafeez et al., 2022b) (Figure 2). There are four clades in insects where the CYP gene can be placed, including CYP2, CYP3, CYP4, and the mitochondrial CYP clade (Nelson, 2011; Zhang et al., 2018). Numerous insect cytochrome P450s (CYPs) have been split into eleven families (Hafeez et al., 2022b). Numerous insect CYPs make up the CYP3 clan of cytochrome P450s, which is further divided into numerous CYP9 family members known to take part in detoxifying processes linked to pesticide resistance (Schuler, 2011; Hafeez et al., 2019). P450 enzymes play an important part in the phase I process of detoxification of many different types of hazardous chemicals, including insecticides, due to their genetic diversity, broad substrate specialization, and catalytic adaptability (Kim et al., 2022). For herbivorous insects, the main mechanism of pesticide resistance is the overexpression of cytochrome P450 detoxifying enzymes (Hafeez et al., 2020b; Wu et al., 2021).

Different P450 enzymes serve different purposes in insects, such as hormone synthesis and regulation, growth and development control, or the digestion of xenobiotic substances (Zhou et al., 2010; Nelson et al., 2013). Due to their central function in pesticide metabolism, cytochrome P450s are frequently implicated in the development of insect resistance to these chemicals (Guo et al., 2012; Panini et al., 2016). One of the most prevalent processes by which insect pests develop resistance to synthetic insecticides is the upregulation of certain detoxifying P450 enzymes within the insect (Nelson, 2011; Hafeez et al., 2022a; Luo et al., 2022). Because of this, insecticide resistance may develop as a result of changes in the activity of detoxifying enzymes in insects in response to exposure to pesticides. Many insect orders, including Coleoptera, Diptera, Hemiptera, Hymenoptera, and Lepidoptera, have shown evidence of P450 overexpression leading to increased resistance to pesticides (Bass et al., 2011; Johnson et al., 2012; Liang et al., 2015; Wang et al., 2015; Chen et al., 2017; Khan et al., 2021a; Khan et al., 2021c; Hafeez et al., 2022a; Siddiqui et al., 2022b).

Invasive species, including *B. tabaci*, *S. exigua*, *S. invicta*, *P. xylostella, etc.*, have been demonstrated to exhibit extremely high levels of resistance to several kinds of insecticides, and this resistance has been linked to CYP detoxifying enzymes (Lai and Su, 2011; Yu et al., 2015; Chen and Zhou, 2017; Ahmad et al., 2018; Wang et al., 2018b; Xie et al., 2018; Hafeez et al., 2019; 2020a; 2020b; 2022c; Siddiqui et al., 2022b). Increased transcriptional levels of CYP6AE97, CYP321A9, CYP9A105, CYP321A16, and CYP459 in the midgut of *S. exigua* larvae treated with different insecticides are just some examples of how *S. exigua* has developed a high level of resistance against several types of insecticides (Wang et al., 2018a; Hu et al., 2019). Moreover, the detoxification mechanism by P450 was reported in various studies. For instance, in *S. invicta* belonging to the order Hymenoptera, CYP was involved in the detoxification of fluralaner (Xiong et al., 2022) (Xiong et al., 2022), fipronil and beta cypermethrin (Siddiqui et al., 2022b). Similarly, in the order Diptera, *D*. *melanogaster*, *C. quinquefasciatus* and *Anopheles funestus* (Giles) (Daborn et al., 2002; Wondji et al., 2009; Itokawa et al., 2010), in the order Hemiptera, *M. persicae* were all reported for the amplification of P450 and insecticide resistance (Puinean et al., 2010). The lambda-cyhalothrin detoxification studies reported the overexpression of P450 in *H. zae* and *H. armigera* (order Lepidoptera) (Li et al., 2000a; 2000b; Chen et al., 2017; Hafeez et al., 2020b). Based on these results, it appears that invasive insects rely heavily on the overexpression of P450 genes in order to detoxify xenobiotics from their bodies.

##### 2.1.5.4 Microbial resistance

Insects host a diverse microbial population that responds to environmental stresses in a dynamic way (Zhang J. et al., 2022). Similar to the insect, the associated microbiota are shaped by the process of natural selection. Changes in food scarcity and diet chemical exposure can all affect its makeup (Adair and Douglas, 2017; Akami et al., 2022). Host microbiota may help the host metabolise pesticides if the host is subjected to selection pressure from these chemicals. It is possible that this mutation is what makes the host less vulnerable to pesticides (Akami et al., 2019a; 2019b). Bacteria capable of breaking down pesticides have been found in numerous natural environments and in several insect orders, including Coleoptera (Akami et al., 2019b), Hemiptera (Kikuchi et al., 2012), Diptera (Cheng et al., 2017; Hassan et al., 2020), and Lepidoptera (Ramya et al., 2016; Almeida et al., 2017). There has been evidence that resistant strains of bacteria from the gut of *P. xylostella* (Xia et al., 2018) and *S. frugiperda* (Almeida et al., 2017) can break down many pesticides (Gomes et al., 2020). Strains of *S. frugiperda* were chosen because of their ability to degrade pesticides; however, these bacteria were lacking in the microbiota of susceptible, unselected larvae (Almeida et al., 2017) (Figure 2).

Insects have a complex defense system built into their digestive tracts, and this system is most likely the driving force behind the organization of gut microbiota (Siddiqui et al., 2022a). Various processes in this defense system influence the host’s tolerance and resistance to bacteria in the insect gut. Though tolerance refers to the capacity to mitigate the detrimental effects of a particular bacterial burden on the host’s health, resistance refers to the capability to lower the bacterial burden to the point where it is no longer a threat to the host’s vigor (Schneider and Ayres, 2008). Insects harboring more bacteria in their digestive systems are more tolerant to other foreign microbes and have less resistance to them than those with less diverse bacterial communities. Because of this, the systems governing gut immunity in various insects might be personalized to the host’s individual needs. Little is known about the systems that mediate tolerance, despite the fact that resistance mechanisms have been the primary focus of immunology studies (Figure 2) (Engel and Moran, 2013). However, digestive tract microbe-host interactions are frequently mutualistic or commensalism in nature. These may be of special importance to the host in terms of minimizing any harmful effects of the resident microbiota on the host.

The insects have a symbiotic microbiome in their guts that aids detoxification (Jing et al., 2020). The enzymatic detoxification method, used by invading insects and the intestinal microbiota responsible for secreting such digestive enzymes, may be used to establish a viable resistance development strategy (Figure 2). Developing resistance in a collection of organisms at the same time is undoubtedly difficult (Barbosa and Levy, 2000). Mutualistic symbiosis plays a significant role in this process because of its synergy and combined powers; the mutualistic alliance formed by two or more species adjusts to adverse conditions (such as pesticide exposure) more quickly than the individual partners of the mutualistic partnership do (Nobre and Aanen, 2012). Accordingly, any beneficial directional flow of resistance development in the system is mitigated or reversed by this adaptability.

The generation turnover of symbiotic microorganisms may overcome the barrier of invasive ants’ extended generation time, allowing for more favorable mutations or gene regulation/alteration, which could lead to pesticide resistance development. Few studies on invasive ants have provided evidence of the ants’ ability to metabolize xenobiotic compounds such as lignin, plant allele chemicals, and pesticides (Engel and Moran, 2013; Siddiqui et al., 2022b; c). Three partiti-like viruses isolated from the African armyworm (*Spodoptera exempta*) have been shown to increase resistance to nucleopolyhedrovirus, while the polydnavirus from parasitoid wasps can disrupt the host insect’s immune system to make sure the survivability of wasp progeny (Strand and Burke, 2013; Xu et al., 2020). Lower termites, which feed primarily on wood, require symbiotic flagellates to break down lignocelluloses and methanogenic archaea to produce methane (Ohkuma, 2008; Shi et al., 2015).

Insect microorganisms can modulate insect resistance to synthetic insecticides through direct breakdown and by stimulating the host’s detoxifying enzymes or immune system (Liu and Guo, 2019; Zhao et al., 2022). For example, the 16S rRNA gene sequencing data demonstrated a decrease in the number of the genera Enterococcus and Stenotrophomonas following polymyxin B therapy, which affected the survival rate of *Bombyx mori* subjected to chlorpyrifos. The host tolerance to chlorpyrifos was improved when germ-free silkworms were given *S. maltophilia*. This bacteria increases host acetylcholinesterase activity but cannot directly break down chlorpyrifos in the stomach (Du et al., 2020). *Aeromonas hydrophila*, an intestine bacteria, was found in substantially greater abundance in deltamethrin-resistant individuals of *Culex pipiens*. After antibiotics were used to clear the stomach of the resistant strains, the resistance level dropped by 66%, and the host’s cytochrome P450 monooxygenase (CYP450) enzyme function dropped by 58%. The resistance and CYP450 enzyme activities were recovered when *A. hydrophila* was supplied, suggesting that *A. hydrophila* promotes host resistance to deltamethrin by boosting CYP450 activity (Xing et al., 2021). Further, *P. xylostella* intestinal *Enterococcus* sp. Upregulates the appearance of an antimicrobial peptide called gloverin, which contributes to the insect’s resistance to the pesticide chlorpyrifos (Xia et al., 2018). *Wolbachia* proliferated in *N. lugens* after treatment with imidacloprid, and their removal decreased CYP450 enzyme activities and NlCYP4CE1 transcript levels. This finding supported the hypothesis that *Wolbachia* increases host resistance to imidacloprid by increasing the expression of the gene encoding the enzyme responsible for its metabolism, NlCYP4CE1 (Cai et al., 2021). The gut microbiome of pollinators like the honeybee (*Apis mellifera*) increases tolerance to pesticides like thiacloprid, tau-fluvalinate, and flumethrin by promoting the expression of genes involved in immunity and detoxification (Wu et al., 2020; Yu et al., 2021). When creating novel pest control methods or reducing pests’ vector competence, it is important to keep an eye on the role of the pests’ microbial partners. Bioremediation and the reduction of xenobiotic toxicity may be greatly aided by the discovery of insect-associated microorganisms capable of detoxifying hazardous chemicals (Mahapatro, 2017).

#### 2.1.6 Resistance-inducing operational factors

The resistance arises from operational factors, including the increased frequency of pesticide applications, the intensive use of insecticides with increased dosage, decreased yields because of pests, and environmental damages (Georghiou and Taylor, 1986). In addition, these factors include a low economic threshold, the repetition of the same insecticide use across multiple generations, the treatment of a large geographical area, the absence of a place of refuge, the use of long-lasting, slow-release formulations of the same insecticide, and the use of insecticides that are chemically similar to those previously used (Sarwar and Salman, 2015; Subramanyam and Hagstrum, 2018).

## 3 Challenges in control of invasive species

Increased global commerce and tourism, climate change, difficulties in protecting borders, Internet commerce, as well as other ways, and invading insects are among global concerns that impose a high economic cost with no apparent remedies in sight (Mooney and Hofgaard, 1999; Lester and Keall, 2005; Venette et al., 2021).

Managing invasive species is challenging because they are often hard to find, and the damage they do may not be noticed for a while. Another challenge is developing new strategies to detect or manage invasive species. Frequently traditional management practices are being used to manage new species, but our ability to control a new invasive species often requires developing novel tools. The last challenge is funding for invasive species control, where there is less support for interdiction activities and extremely high expenditures to launch large-scale actions against established species. Most efforts to control and study invasive species fall into two phases. The initial phases of an invasion (forecast and prevention) give way to later phases (early detection, fast response, mitigation, and management) as the invasion moves forward through the arrival, settlement, and spreading phases (Venette et al., 2021). Cost-benefit evaluations show that focusing on prevention and containment rather than damage control and ecosystem restoration is the best way to deal with invasive species (Leung et al., 2002; Lampert and Liebhold, 2021). Sociologists term the spread of alien invasive species a “wicked” problem because of the complex interplay between its many root causes (such as globalization, climate change, public ignorance, and inadequate biosecurity measures) (McNeely, 2013; Venette and Morey, 2020). Researchers may do their part to control invasive species by focusing on objectives that have widespread support and can be measured quantitatively. The costs and advantages of a proposed solution (such as a new sample plan or control system) should be clearly stated and compared to the *status quo*. Multiple reasons have led to an increase in the number of invasive species that have migrated over political and natural boundaries, necessitating a multifaceted approach to eradicating them.

## 4 Management strategies

The best strategy for combating exotic species is based on prediction and prevention. The goal is straightforward: predict which species or pathways provide unacceptable dangers and prevent them from entering a target area. Such a plan necessitates a defined indicator of success. As the number of alien arthropods keeps rising, every new invasion could be seen as a breach in biosecurity (Finch et al., 2021). However, international trade has expanded faster than new species have been introduced. This pattern implies biosecurity measures have been relatively effective (Venette and Morey, 2020) but are distant from an accurate degree of success (Saccaggi et al., 2016).

To determine whether or not a product can be legally imported, how surveillance programs should be set up, and what actions should be taken after an invasive species has been discovered in a sensitive area, biosecurity professionals will continue to rely on spatially-explicit pest risk assessments (Venette et al., 2010). Locations where an invasive species is most likely to cause damage, are described, emphasizing the environmental factors that must be present for a pest to establish. However, methods for creating these evaluations that are credible, expandable, and cost-effective are required.

Particularly in machine learning and other statistical models, the difficulty in creating models that can be successfully transferred to new space and time is becoming more widely acknowledged as a conceptual barrier (Morey and Venette, 2020). While process-based models appear promising, the sheer volume of information needed to apply them to the hundreds or even thousands of species of importance makes them impractical (Venette, 2017). Improvements in the ability to record shifts in insect population abundance, distribution, and phenology directly result from the democratization of data collecting made possible by the rise of mobile apps and open-access databases. To strengthen predictions, advances in phylogeography will allow for a more thorough accounting of invasions, the identification of invasive phenotypes, and the explicit consideration of genotype x environment relationships (Estoup and Guillemaud, 2010; Roe et al., 2019).

Early detection surveys can be more efficiently planned with the help of pest risk maps, which identify probable invasion or damage regions. Our current capacity for surveying the endangered area may be inadequate (Venette et al., 2010). More study is required, in our opinion, to determine the likelihood of finding low concentrations of invasive arthropods using a given sample strategy (Sandercock et al., 2022). Experts devised a “risk-based monitoring” method that concentrates its sampling efforts in areas with the greatest density of the invasive pest preferred and most economically valuable host plants (Prattley, 2009). As a result, we can lower the expense of detecting invasive species, while additional studies are needed to determine whether or not this method is effective for invading insects. It can be computationally challenging to utilize the strategies pioneered by Yemshanov et al. (2017, Yemshanov et al. 2019) to optimize the spatial distribution of sampling efforts.

When combined with conventional taxonomical knowledge, the recent advancements in genomes provide a method for quick validation of species identification. Additionally, genomic techniques allow for the differentiation of strains, haplotypes, or biotypes within a species (Venette et al., 2021). To better depict the geographic origins of an invading population, specific haplotype data can be used, as shown for the *S. frugiperda* (Goergen et al., 2016), *H. halys* (Xu et al., 2014), and walnut twig beetle (*Pityophthorus juglandis*) (Rugman-Jones et al., 2015). Precise pest origin information allows for early country selection in the search for natural enemies, typically species-specific parasitoids, which may then be released in invaded nations after being evaluated for safety and efficacy (Wyckhuys et al., 2020; Venette et al., 2021).

Following the initial settlement and expansion of invasive species, it is prudent to invest immediately in R&D to promote integrated pest management (IPM) strategies. IPM is “an ecosystem-based strategy that focuses on long-term prevention of pests or their damage through a combination of techniques such as biological control, habitat manipulation, modification of cultural practices, and use of resistant varieties (Sujatha et al., 2022). Pesticides are used only after monitoring indicates they are needed according to established guidelines, and treatments are made to remove only the target organism (Singh et al., 2018). Pest control materials are selected and applied to minimize risks to human health, beneficial and non-target organisms, and the environment” (IPM, 1996). These decision-making guidelines are applied to pest management on a regional scale rather than just on a farm or a piece of land. Ironically, many cropping systems already have IPM in place for indigenous pests. In the case of *H. halys* in the U.S. apple crop, for example, >$37 million was wasted related to destruction and elevated insecticide application costs because of the advent of the pest (Leskey and Nielsen, 2018). IPM solutions for invasive species often involve short- and long-term plans, just as they do when dealing with endemic pests. Pesticidal treatment is frequently prioritized first since it is effective at maintaining growers’ capital, although agreements to fund research into biocontrol, pest-resistant cultivars, and cultural measures (Radcliffe et al., 2009), physical exclusions (Rogers et al., 2016), and “attract and kill” behavior-based insect traps (Gregg et al., 2018). Developing resistance to invasive pests is becoming increasingly crucial for trees and perennial crops. Innovative technologies, such as transgenic insecticidal plants or genetic biocontrol agents facilitated by gene drives, have the potential to either complement or replace IPM programs, depending on whether or not they are granted the necessary regulatory permissions (Hutchison et al., 2010) (Maselko et al., 2017; Sudweeks et al., 2019).

## 5 Future implications

Insect invasions caused by globalization have posed a severe danger to native plant and animal life, with some species becoming extinct. Because of increased international trade, more seeds and other planting materials are being transported worldwide, increasing the risk of invasive pests being introduced into new habitats and countries.

As a matter of biosecurity, it is recommended that invasive species be identified as soon as possible to control them from invading new regions. However, many underdeveloped nations are particularly lagging in early detection. Without natural predators and parasites, invasive species in their new environment can quickly spread and cause severe damage to economically important plant species and biodiversity.

Strategies to prevent or lessen the impact of future incursions should be part of any future approach to managing invasive species. It is possible to reduce the likelihood of introducing new pest species into an area if people have a general knowledge of invasive species and work together globally by sharing data about these organisms and the predators and parasites that threaten them. The increased science-based knowledge, innovation, and expertise in managing invasive species helped control them more efficiently.

The local flora and fauna, agriculture, horticulture, and the environment severely impact the harmful impacts of invasive pest species. These species negatively impact biodiversity and may also affect the nation’s economy. Scientists must work together across areas in order to detect invasive pests and analyze their ecological problems, environmental risks in different habitats, financial damage, and management alternatives. This can be done by creating generic, particular, and context-dependent action plans. This highlights the significance of the quarantine in preventing the spread of destructive alien pests.
